# Mental Health Service Utilization Rates Among Commercially Insured Adults in the US During the First Year of the COVID-19 Pandemic

**DOI:** 10.1001/jamahealthforum.2022.4936

**Published:** 2023-01-06

**Authors:** Ryan K. McBain, Jonathan Cantor, Megan F. Pera, Joshua Breslau, Dena M. Bravata, Christopher M. Whaley

**Affiliations:** 1 RAND Corporation, Boston, Massachusetts; 2 RAND Corporation, Santa Monica, California; 3 Castlight Health, San Francisco, California; 4 RAND Corporation, Pittsburgh, Pennsylvania

## Abstract

**Importance:**

The COVID-19 pandemic has been associated with an elevated prevalence of mental health conditions and disrupted mental health care throughout the US.

**Objective:**

To examine mental health service use among US adults from January through December 2020.

**Design, Setting, and Participants:**

This cohort study used county-level service utilization data from a national US database of commercial medical claims from adults (age >18 years) from January 5 to December 21, 2020. All analyses were conducted in April and May 2021.

**Main Outcomes and Measures:**

Per-week use of mental health services per 10 000 beneficiaries was calculated for 5 psychiatric diagnostic categories: major depressive disorder (MDD), anxiety disorders, bipolar disorder, adjustment disorders, and posttraumatic stress disorder (PTSD). Changes in service utilization rates following the declaration of a national public health emergency on March 13, 2020, were examined overall and by service modality (in-person vs telehealth), diagnostic category, patient sex, and age group.

**Results:**

The study included 5 142 577 commercially insured adults. The COVID-19 pandemic was associated with more than a 50% decline in in-person mental health care service utilization rates. At baseline, there was a mean (SD) of 11.66 (118.00) weekly beneficiaries receiving services for MDD per 10 000 enrollees; this declined by 6.44 weekly beneficiaries per 10 000 enrollees (β, –6.44; 95% CI, –8.33 to –4.54). For other disorders, these rates were as follows: anxiety disorders (mean [SD] baseline, 12.24 [129.40] beneficiaries per 10 000 enrollees; β, –5.28; 95% CI, –7.50 to –3.05), bipolar disorder (mean [SD] baseline, 3.32 [60.39] beneficiaries per 10 000 enrollees; β, –1.81; 95% CI, –2.75 to –0.87), adjustment disorders (mean [SD] baseline, 12.14 [129.94] beneficiaries per 10 000 enrollees; β, –6.78; 95% CI, –8.51 to –5.04), and PTSD (mean [SD] baseline, 4.93 [114.23] beneficiaries per 10 000 enrollees; β, –2.00; 95% CI, –3.98 to –0.02). Over the same period, there was a 16- to 20-fold increase in telehealth service utilization; the rate of increase was lowest for bipolar disorder (mean [SD] baseline, 0.13 [16.72] beneficiaries per 10 000 enrollees; β, 1.40; 95% CI, 1.04-1.76) and highest for anxiety disorders (mean [SD] baseline, 0.20 [9.28] beneficiaries per 10 000 enrollees; β, 9.12; 95% CI, 7.32-10.92). When combining in-person and telehealth service utilization rates, an overall increase in care for MDD, anxiety, and adjustment disorders was observed over the period.

**Conclusions and Relevance:**

In this cohort study of US adults, we found that the COVID-19 pandemic was associated with a rapid increase in telehealth services for mental health conditions, offsetting a sharp decline in in-person care and generating overall higher service utilization rates for several mental health conditions compared with prepandemic levels.

## Key Points

**Question:**

Did mental health service utilization in the US change for different psychiatric diagnoses during the first year of the COVID-19 pandemic (January to December 2020)?

**Findings:**

In this cohort study of 5 142 577 commercially insured adults across all 50 US states, the weekly rate of in-person mental health service utilization decreased by more than 50% after the start of the COVID-19 pandemic but concurrent increases in telehealth led to a slight increase in total utilization for anxiety disorders and stability in total volume of service for other disorders.

**Meaning:**

The findings suggest that the pandemic disrupted in-person mental health care but expansion of telehealth enabled care for an increasing number of patients.

## Teaser

This cohort study examines changes in use of in-person and telehealth mental health services among commercially insured adults across all 50 US states during the first year of the COVID-19 pandemic.

## Introduction

In a typical year, 1 in 5 US adults are living with a mental illness.^1^ Of these 50 million individuals, only half received mental health services from 2020 to 2021 due in large part to workforce and other capacity constraints.^2^ Although treatment prevalence for mental disorders such as depression and anxiety has been gradually increasing,^3,4^ the global COVID-19 pandemic has risked disrupting these trends in at least 3 ways. First, the prevalence of mental disorders increased significantly from 2020 to 2021.^5,6,7^ The pandemic has been associated with decreased social interactions and physical exercise,^8,9,10^ threats to individuals’ employment and economic security,^11^ and an escalation in morbidity and mortality^12^—all of which are likely to have contributed to widespread psychological distress.^13,14,15^

Second, the ability of health facilities to support in-person, nonemergency care contracted, particularly during the early months of the pandemic.^16^ A survey conducted by the National Council for Mental Well-being in April 2020 found that 93% of responding behavioral health organizations throughout the US reported reduced operations, including 47% furloughing or discharging employees as a result of the COVID-19 pandemic.^17^ This survey was conducted after publication of Centers for Disease Control and Prevention (CDC) recommendations for enhanced precautions for nonemergency health care^18^ as well as shelter-in-place ordinances.^19^ Electronic medical record systems and claims data have also revealed substantial declines in emergency department visits and hospitalizations,^20^ although the picture for mental health services has been more nuanced, with more recent evidence indicating elevated emergency department visit rates for mental health conditions between March and October 2020.^21,22^

Third, health systems have promoted rapid expansion of telehealth services,^23,24^ including the provision of outpatient mental health care.^25^ This transition was bolstered by legislative mandates requiring private insurers to cover telemental health care and the creation of new Centers for Medicare & Medicaid Services reimbursement codes.^26,27^ One recent study found a 20-fold increase in the incidence of telemedicine utilization,^28^ while another found that over 50% of adults receiving behavioral health services from March to May 2020 had done so through teleconference or videoconference.^29^

Against the backdrop of these trends, it remains unclear how mental health service utilization for specific conditions at a national level has evolved over the course of the pandemic compared with prepandemic levels. Previous studies have examined trends in mental health care utilization during the COVID-19 pandemic by Medicare and Medicare Advantage beneficiaries and veterans^30,31,32^ and the perspective of mental health professionals offering telehealth during the COVID-19 pandemic.^33^ In this study, we analyzed weekly mental health service utilization rates at the US county level among commercially insured adults across all 50 states from January 5 through December 21, 2020, according to 5 diagnostic categories: major depressive disorder, anxiety disorders, bipolar disorder, adjustment disorder, and posttraumatic stress disorder (PTSD). Additionally, we disaggregated analyses according to patient sex, age group, and mode of service delivery.

## Methods

### Study Design and Participants

In this cohort study, to describe trends in mental health service utilization before vs after the onset of the COVID-19 pandemic in the US, we examined weekly medical claims from commercially insured adults (age >18 years) between January 5 and December 21, 2020. Medical claims information was provided by Castlight Health, a health benefits manager for employer-sponsored health insurance plans for approximately 200 employers across all 50 states.^34^ We limited our analysis to adults as the service use patterns, prevalence, and sources of care are liable to differ between children and adults. This study was approved by the RAND Human Subjects Protection Committee, which waived informed consent because data were deidentified. The study followed the Strengthening the Reporting of Observational Studies in Epidemiology (STROBE) reporting guideline.

### Measures

Medical claims were classified according to the *International Statistical Classification of Diseases and Related Health Problems, Tenth Revision (ICD-10)* primary diagnosis codes for mental disorders at the 3- and 4-digit level to preserve sufficient cell size for anonymity. These included major depressive disorder (F32, F33), anxiety disorders (F40, F41), bipolar disorder (F31), adjustment disorder (F43.2), and PTSD (F43.1).^35^ We did not include schizophrenia or schizoaffective disorder as the number of individuals within the data set who received these diagnoses was too small to merit inspection. We calculated the weekly number of individuals per 10 000 eligible beneficiaries at the US county level who received services for which each of these code categories was assigned. To ensure that estimates did not reflect dropouts or those who lost coverage during the pandemic, we limited eligible beneficiaries to those with continuous enrollment throughout the duration of the study period. We identified the interval from January 5 to March 13, 2020, the date on which COVID-19 was declared a national public health emergency, as the pre–COVID-19 interval^36^ and dates following March 13 as the post–COVID-19 interval.

To inspect demographic differences in service utilization rates, we generated separate service utilization rates according to patient age category (19-26, 27-45, 46-59, and ≥60 years) and sex (female, male) and mode of service delivery (telehealth, in-person). In addition, we linked the patient address to county-level demographic characteristics, including the share (percentage) of the adult population that was Black, Hispanic, or White, as indexed in the American Community Survey^37^; weekly prevalence of SARS-CoV-2 infection cases per 10 000 population, as recorded by USAFacts^38^; monthly unemployment rates, as reported by the US Bureau of Labor Statistics^39^; and county-level rurality, as recorded by the US Department of Agriculture from census data.^40^

### Statistical Analysis

To examine the association between mental health service utilization and the onset of the COVID-19 pandemic in the US, we used longitudinal, multivariable fixed-effects regression analyses to estimate change in utilization rates in the weeks following the national emergency declaration compared with prior weeks. In addition to a dichotomous time variable for the national public health emergency declaration, we included the following covariates: patient age and sex, county-level rurality, racial and ethnic composition, monthly unemployment rate, and weekly SARS-CoV-2 infection cases per 10 000 population. Fixed effects were included for calendar week and US state. Standard errors were clustered at the state level. Separate regressions were estimated for each mode of service: in-person and telehealth. We also investigated whether changes in mental health service utilization rates that occurred following the onset of the pandemic varied according to patient age group and sex. These models included the same fixed effects described above but also examined interactions between the dichotomous time variable and the demographic characteristic categories.

Lastly, to examine changes in service utilization over the course of the COVID-19 pandemic, we executed an alternative model specification: a mixed-effects, segmented regression. In this framework, we included separate intercept and slope terms for the pre–COVID-19 and post–COVID-19 periods. This allowed us to examine whether time trends in service utilization differed between intervals as well as whether there was a sudden shift in service utilization following the March 13 national emergency declaration. The preperiod contained 10 time points, while the postperiod contained 40 time points, an interval deemed sufficient for segmented regression.^41^ Analyses were conducted in April and May 2021. All precision estimates are reported using 2-sided 95% CIs. Analyses were performed in Stata, version 15.1 (StataCorp LLC).^42^

## Results

The study included 5 142 577 commercially insured adults. Prior to the national public health emergency declaration (January 5 to March 13, 2020), mean (SD) weekly mental health service utilization rates among adults ranged from 4.83 (0.27) per 10 000 enrollees for bipolar disorder to 23.28 (1.08) per 10 000 enrollees for anxiety disorders (Table 1). From March 14 through December 20, 2020, service utilization rates increased by 8.0% across the 5 diagnostic categories, ranging from 6.4% for severe stress or adjustment disorders to 14.0% for anxiety disorders. Table 2 shows details of service utilization rates according to period, diagnostic category, mode of service delivery, and patient sex.

**Table 1.** Mental Health Service Utilization Rates by Mode of Service Delivery

Diagnostic group	Utilization rate, mean (SD)^a^								
	In-person			Telehealth			Total		
	Pre–COVID-19	Post–COVID-19	Difference, %	Pre–COVID-19	Post–COVID-19	Difference, %	Pre–COVID-19	Post–COVID-19	Difference, %
Major depressive disorder	16.97 (2.04)	7.24 (0.69)	−57.3	0.61 (1.03)	11.13 (0.97)	1724.6	17.59 (1.07)	18.37 (1.38)	4.4
Anxiety disorders	22.24 (2.69)	9.95 (0.95)	−55.3	1.04 (1.71)	16.59 (1.49)	1495.2	23.28 (1.08)	26.54 (2.19)	14.0
Bipolar disorder	4.67 (0.53)	2.24 (0.28)	−52.0	0.16 (0.29)	3.24 (0.26)	1925.0	4.83 (0.27)	5.48 (0.42)	13.5
Adjustment disorders	21.46 (2.77)	9.73 (0.97)	−54.7	0.74 (1.32)	14.30 (1.47)	1832.4	22.20 (1.53)	24.03 (2.21)	8.2
PTSD	3.79 (0.42)	1.64 (0.14)	−56.7	0.14 (0.25)	2.54 (0.24)	1714.3	3.93 (0.18)	4.18 (0.32)	6.4

Abbreviation: PTSD, posttraumatic stress disorder.

^a^ The pre–COVID-19 service utilization rate was defined as weekly beneficiaries receiving services for mental health conditions per 10 000 enrollees from January 5 through March 12, 2020. The post–COVID-19 service utilization rate was defined as weekly beneficiaries receiving services for mental health conditions per 10 000 enrollees from March 13 through December 21, 2020. Standard deviations represent week-over-week variation within the period of interest.

**Table 2.** Mental Health Service Utilization Rates by Sex

Diagnostic group	Utilization rate, mean (SD)^a^								
	Female			Male			Total		
	Pre–COVID-19	Post–COVID-19	Difference, %	Pre–COVID-19	Post–COVID-19	Difference, %	Pre–COVID-19	Post–COVID-19	Difference, %
Major depressive disorder	23.98 (1.44)	25.51 (1.94)	6.0	11.41 (0.74)	11.45 (0.85)	0.3	17.59 (1.07)	18.37 (1.38)	4.4
Anxiety disorders	31.50 (1.47)	37.08 (3.12)	17.7	15.32 (0.75)	16.32 (1.33)	6.5	23.28 (1.08)	26.54 (2.19)	14.0
Bipolar disorder	5.46 (0.25)	5.92 (0.46)	8.4	2.45 (0.15)	2.48 (0.20)	1.2	4.83 (0.27)	5.48 (0.42)	13.5
Adjustment disorders	28.61 (2.12)	31.96 (3.14)	11.7	16.00 (0.99)	16.35 (1.35)	2.2	22.20 (1.53)	24.03 (2.21)	8.2
PTSD	7.64 (0.39)	8.78 (0.66)	14.9	2.11 (0.15)	2.28 (0.20)	8.1	3.93 (0.18)	4.18 (0.32)	6.4

Abbreviation: PTSD, posttraumatic stress disorder.

^a^ The pre–COVID-19 service utilization rate was defined as weekly beneficiaries receiving services for mental health conditions per 10 000 enrollees from January 5 through March 12, 2020. The post–COVID-19 service utilization rate was defined as weekly beneficiaries receiving services for mental health conditions per 10 000 enrollees from March 13 through December 21, 2020. Standard deviations represent week-over-week variation within the period of interest.

As shown in the Figure, service utilization rates declined by 52% to 57% for in-person care following the US national emergency declaration. For example, mean (SD) service utilization for depression care declined from 17.0 (2.0) beneficiaries per 10 000 members per week to 7.2 (0.7) beneficiaries per 10 000 members per week, a 57% decline. Lower rates of in-person service utilization also persisted through December, the last month for which claims data were available. By contrast, utilization rates of telehealth services increased across all diagnostic categories following the national declaration by a factor of 16 to 20 (ie, an increase of 1495.2% to 1925.0%). Unadjusted trends—both declines in in-person care and increases in telehealth—were statistically significant in all instances. Total service utilization from March through December 2020 was higher than prior to COVID-19 for anxiety disorders (β, 3.52; 95% CI, 0.87-6.17), but the difference was not statistically significant for other diagnostic categories. When examining unadjusted trend differences between males and females, we also found that increases in the rate of weekly service utilization (before vs after March 13) were larger for females than for males among those receiving services for anxiety disorders (β, 2.99; 95% CI, 0.48-5.51). Differences were not statistically significant for the remaining diagnostic categories.

**Figure.**
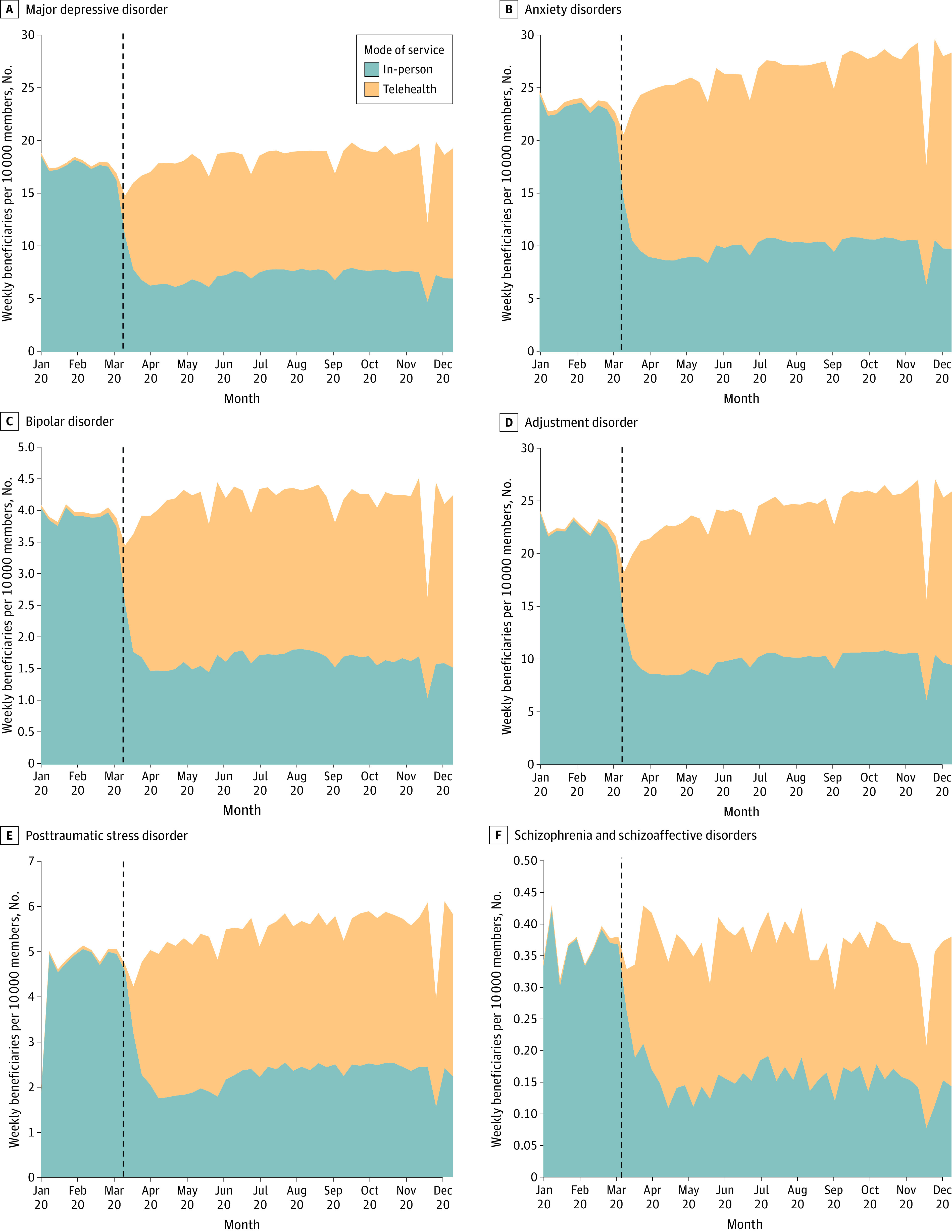
Mental Health Utilization Rates in the US by In-Person vs Telehealth Service The full date range was January 5 to December 21, 2020. The COVID-19 national emergency was declared on March 13, 2020 (dashed vertical lines).

Multivariable regression models were used to inspect trends in total service utilization and in-person vs telehealth service utilization by diagnostic category after adjusting for patient- and county-level characteristics. Looking at total service utilization, we found only 1 significant difference between the 2 periods; there was a higher rate of treatment for anxiety disorders following the national declaration (baseline: mean [SD] 12.44 [129.83] beneficiaries per 10 000 enrollees; β, 3.79; 95% CI, 1.19-6.38; *P* = .005). In interaction models that examined the associations between changes in total service utilization and patient sex and age, we found that uptake was greater for females seeking services for anxiety disorders (β, 3.01; 95% CI, 0.50-5.52; *P* = .02) and lower for older adults (aged ≥60 years) seeking services for adjustment disorders (β, −3.86; 95% CI, −6.67 to −1.05; *P* = .008) (Table 3).

**Table 3.** Service Utilization Rates per 10 000 Enrollees Before vs After the COVID-19 National Emergency Declaration^a^

Characteristic	β (95% CI)^b^				
	Depressive disorder	Anxiety disorders	Bipolar disorder	Adjustment disorder	PTSD
COVID-19 pandemic national declaration^c^	−0.85 (−3.58 to 1.88)	2.47 (−1.42 to 6.36)	−0.24 (−1.27 to 0.80)	−0.49 (−3.22 to 2.23)	1.61 (−0.41 to 3.63)
**Individual level**					
Sex					
Female	10.02 (7.77 to 12.26)	11.72 (8.89 to 14.55)	3.05 (2.08 to 4.02)	6.45 (4.25 to 8.64)	5.33 (3.45 to 7.21)
Male	1 [Reference]	1 [Reference]	1 [Reference]	1 [Reference]	1 [Reference]
Age group, y					
19-26	1 [Reference]	1 [Reference]	1 [Reference]	1 [Reference]	1 [Reference]
27-45	−4.84 (−8.60 to −10.7)	−8.93 (−11.61 to −6.24)	−0.82 (−2.23 to 0.59)	2.11 (−1.23 to 5.45)	2.56 (−0.08 to 5.20)
46-59	−9.10 (−13.22 to −4.97)	−10.89 (−15.32 to −6.45)	−1.90 (−3.29 to −0.51)	−0.73 (−4.02 to 2.56)	−0.52 (−2.37 to 1.33)
≥60	−11.26 (−14.93 to −7.30)	−17.64 (−21.06 to 14.22)	−3.03 (−4.30 to −1.75)	−3.91 (−7.19 to −0.64)	−3.07 (−4.25 to −1.89)
**County level**					
Female × national declaration	1.45 (−0.25 to 3.14)	3.01 (0.50 to 5.52)	0.24 (−0.55 to 1.04)	1.68 (−0.03 to 3.40)	0.79 (−0.51 to 2.07)
Age 27-45 y × national declaration	−0.79 (−3.62 to 2.04)	3.13 (−0.26 to 6.52)	−0.20 (−1.42 to 1.03)	1.31 (−2.04 to 4.66)	−1.62 (−3.83 to 0.59)
Age 46-59 y × national declaration	−0.21 (−2.85 to 2.44)	−2.35 (−5.91 to 1.22	−0.63 (−1.68 to 0.41)	−2.24 (−4.88 to 0.39)	−1.74 (−3.44 to −0.04)
Age ≥60 y × national declaration	−0.35 (−3.03 to 2.34)	−1.36 (−4.53 to 1.81)	−0.35 (−1.21 to 0.52)	−3.86 (−6.67 to −1.05)	−0.95 (−2.36 to 0.45)

Abbreviation: PTSD, posttraumatic stress disorder.

^a^ Fixed-effects regression analysis included additional county-level demographic covariates as well as state and week fixed effects (not shown).

^b^ The β coefficient represents the change in the number of beneficiaries per 10 000 beneficiaries on a weekly basis.

^c^ National emergency declaration represents prepandemic and postpandemic variables for the weeks before vs after March 13, 2020.

In contrast with total service utilization, we observed significant countervailing declines and increases in service utilization of in-person and telehealth care, respectively. With regard to in-person care, we observed decreases in weekly beneficiaries per 10 000 enrollees for major depressive disorder (baseline mean [SD], 11.66 [118.00] beneficiaries per 10 000 enrollees; β, −6.44; 95% CI, −8.33 to −4.54; *P* < .001), anxiety disorders (baseline mean [SD], 12.24 [129.40] beneficiaries per 10 000 enrollees; β, −5.28; 95% CI, −7.50 to −3.05; *P* < .001), bipolar disorder (baseline mean [SD], 3.32 [60.39] beneficiaries per 10 000 enrollees; β, −1.81; 95% CI, −2.75 to −0.87; *P* < .001), adjustment disorders (baseline mean [SD], 12.14 [129.94] beneficiaries per 10 000 enrollees; β, −6.78; 95% CI, −8.51 to −5.04; *P* < .001), and PTSD (baseline mean [SD], 4.93 [114.23] beneficiaries per 10 000 enrollees; β, −2.00; 95% CI, −3.98 to −0.02; *P* = .04). With regard to telehealth, we observed increases for major depressive disorder (baseline mean [SD], 0.10 [2.94] beneficiaries per 10 000 enrollees; β, 5.97; 95% CI, 4.94-7.00; *P* < .001), anxiety disorders (baseline mean [SD], 0.20 [9.28] beneficiaries per 10 000 enrollees; β, 9.12; 95% CI, 7.32-10.92; *P* < .001), bipolar disorder (baseline mean [SD], 0.13 [16.72] beneficiaries per 10 000 enrollees; β, 1.40; 95% CI, 1.04-1.76; *P* < .001), adjustment disorders (baseline mean [SD], 0.13 [4.02] beneficiaries per 10 000 enrollees; β, 5.95; 95% CI, 4.59-7.32; *P* < .001), and PTSD (baseline mean [SD], 0.10 [5.66] beneficiaries per 10 000 enrollees; β, 2.91; 95% CI, 1.29-4.52; *P* < .001).

Segmented regression analyses were used to model separate intercepts and slopes for both time intervals: before and after the national emergency declaration (Table 4). In the preperiod, we observed stable mental health service utilization rates across all diagnostic categories; none of the pre–COVID-19 slopes differed significantly from 0 except for a modest increase in service utilization for bipolar disorder during this period. Following the national emergency declaration, there was an immediate increase in total service utilization for anxiety disorders (β, 1.62; 95% CI, 0.18-3.06; *P* = .03), as indicated by the post–COVID-19 intercept term in Table 4. This was not observed for other conditions. We also observed gradually increasing total service utilization rates for the remainder of 2020 for major depressive disorder (β, 0.09; 95% CI, 0.04-0.13; *P* < .001), anxiety disorders (β, 0.13; 95% CI, 0.03-0.23; *P* = .01), and adjustment disorders (β, 0.08; 95% CI, 0.02-0.14; *P* = .007), as indicated by the post–COVID-19 slope terms in Table 4. No other differences were statistically significant.

**Table 4.** Segmented Regression Analysis of Service Utilization Rates Before vs After March 13, 2020^a^

Mode of service delivery	β (95% CI)^b^				
	Depressive disorder	Anxiety disorders	Bipolar disorder	Adjustment disorders	PTSD
**In-person service utilization**					
Pre–COVID-19^c^					
Slope	0.01 (−0.15 to 0.17)	−0.14 (−0.32 to 0.04)	0.06 (0.00 to 0.12)	0.07 (−0.12 to 0.25)	0.11 (−0.00 to 0.23)
Post–COVID-19^d^					
Intercept	−3.62 (−4.68 to −2.55)	−3.14 (−4.61 to −1.67)	−1.38 (−2.03 to −0.73)	−4.91 (−6.45 to −3.36)	−1.25 (−2.12 to −0.37)
Slope	0.00 (−0.04 to 0.03)	−0.01 (−0.06 to 0.04)	0.00 (−0.02 to 0.02)	−0.05 (−0.09 to 0.00)	−0.02 (−0.07 to 0.02)
**Telehealth service utilization**					
Pre–COVID-19^c^					
Slope	−0.01 (−0.03 to 0.00)	0.00 (−0.02 to 0.02)	0.01 (0.00 to 0.02)	0.00 (−0.01 to 0.01)	0.00 (0.00 to 0.01)
Post–COVID-19^d^					
Intercept	3.45 (2.37 to 4.52)	4.76 (3.39 to 6.12)	1.08 (0.75 to 1.42)	2.64 (1.61 to 3.67)	1.58 (0.88 to 2.29)
Slope	0.09 (0.06 to 0.12)	0.14 (0.06 to 0.23)	0.01 (0.00 to 0.03)	0.13 (0.09 to 0.17)	0.03 (0.00 to 0.05)
**Total service utilization**					
Pre–COVID-19^c^					
Slope	0.00 (−0.17 to 0.16)	−0.14 (−0.32 to 0.05)	0.07 (0.01 to 0.13)	0.07 (−0.12 to 0.26)	0.12 (0.00 to 0.24)
Post–COVID-19^d^					
Intercept	−1.46 (−2.98 to 0.06)	1.62 (0.18 to 3.06)	−0.29 (−0.95 to 0.36)	−0.98 (−2.44 to 0.48)	0.34 (−0.87 to 1.55)
Slope	0.09 (0.04 to 0.13)	0.13 (0.03 to 0.23)	0.01 (−0.01 to 0.03)	0.08 (0.02 to 0.14)	0.00 (−0.05 to 0.06)

Abbreviation: PTSD, posttraumatic stress disorder.

^a^ Segmented regression included demographic covariates as well as state and week fixed effects. Service utilization rates were defined as the number of weekly beneficiaries using services per 10 000 enrollees.

^b^ The β coefficient represents the change in number of beneficiaries per 10 000 enrollees on a weekly basis.

^c^ The pre–COVID-19 slope represents the week-over-week change in service utilization rates from January 5 through March 12, 2020.

^d^ The post–COVID-19 intercept represents the immediate change in service utilization rates for the week of March 13, 2020, compared with the previous week. The post–COVID-19 slope represents the week-over-week change in service utilization rates from March 13 through March 20, 2020.

## Discussion

This study found large-scale changes in national mental health service utilization rates throughout 2020 in a broad cross section of commercially insured US adults following the onset of the COVID-19 pandemic. We found a greater than 50% decline in in-person service utilization rates across 5 major diagnostic categories, which was entirely offset by rapid expansion in telehealth service utilization. For 3 categories of conditions (anxiety disorders, major depressive disorder, and adjustment disorders), we also found evidence that service utilization rates increased throughout the pandemic compared with before the pandemic’s onset.

Prior studies have chronicled an elevated prevalence of psychological distress and common mental disorders such as anxiety and depression over the course of the pandemic.^14,15^ For example, a recent CDC study estimated that the percentage of US adults reporting anxiety or depressive symptoms in the past 7 days during February 2021 was over 40%.^43^ Examining more than 2 million health care encounters between January and December 2020, we did not observe a comparable increase in service utilization. Rather, we found a gradual increase in service utilization such that, by November and December, utilization rates were roughly 10% to 20% higher than they were in January and February for select diagnostic categories.

Nevertheless, these estimates mask a remarkable transition in the US health system from in-person to virtual care. On the one hand, in-person care for nonemergency services, including outpatient mental health care, rapidly contracted beginning in March 2020, following new guidance from the CDC^44^ and shelter-in-place ordinances passed by governors and mayors.^45^ These findings provide evidence of the long-term outcomes of those policies, with in-person mental health services still being roughly 50% lower at the end of 2020 compared with the beginning of the year. On the other hand, the Department of Health and Human Services relaxed Health Insurance Portability and Accountability Act compliance regulations to promote broader adoption of telehealth,^46^ which was coupled with executive orders and state legislation mandating that commercial health plans reimburse for telehealth services at parity with in-person care.^47^ While preliminary studies documented a marked increase in scale-up of telehealth services,^48^ to our knowledge, this is the first study to show that the magnitude of this increase (roughly a 16- to 20-fold increase in utilization) fully compensated for the decline in in-person care.

For the 3 highest-prevalence diagnostic categories, we also found that service utilization continued to increase throughout 2020, primarily in the telehealth context. This was most apparent among those seeking treatment for anxiety, for which we observed more than a 20% increase in service utilization by the end of the year, as well as an immediate increase in treatment seeking following the national emergency declaration on March 13. These findings are in line with prior evidence indicating that heightened anxiety has been the most commonly documented mental health impact of the pandemic.^43,49^ By contrast, we did not find comparable increases for mental illnesses such as bipolar disorder and PTSD.

Our examination of the demographics associated with mental health service utilization rates had 3 major findings. First, although females had higher utilization rates for several mental health conditions prior to the onset of the pandemic, the increase in service utilization for anxiety disorders throughout the pandemic was larger for females than for males. There may be several explanations for this, such as greater willingness among females compared with males to use telehealth^50^ or stressors that may have disproportionately been placed on females, such as caring for out-of-school children. Second, we found lower service utilization rates in rural counties that were nonadjacent to metropolitan regions. As 1 recent article indicated, this may be a result of telehealth services failing to reach remote, lower-income communities throughout the US.^28^ Third, service utilization rates for adults aged 46 years or older were lower than those among younger adults. While this may be partly attributable to a lower prevalence of certain conditions among older adults,^51^ the consistency of this trend across diagnostic categories suggests that other factors may play a role, including the digital literacy and comfortability of older adults to use videoconferencing for communicating with health care professionals.

### Limitations

This study has several limitations. First, it used data from a subset of individuals with employer-based private insurance. As such, those in the data set predominantly represent employed adults with symptom severity below a threshold at which gainful employment may be inhibited. Our data set did not include those who were publicly insured and was not representative of all commercially insured individuals. Second, we do not know the number or proportion of individuals in the study sample who were continuously insured throughout the study period. Changes in the study sample may be responsible for some of our results. Third, our results are limited to 2020. We were not able to determine whether service utilization rates evolved further in the first months of 2021 due to the lag time of claims data. However, the trends we observed were stable over the 9-month period following the national health emergency declaration. Fourth, we did not evaluate the effects of state-level policies, which may have further influenced mental health care utilization.^19^ Likewise, the national emergency declaration was an important date marking a transition in the US public health system response; however, there was substantial geographic heterogeneity in the onset of the pandemic throughout the nation. Fifth, we were not able to assess quality of care and whether it differed between in-person and telehealth services. Although evidence from previous studies^52,53^ indicates that telemental health services are comparable with in-person services for conditions such as anxiety and depression, more evidence is required to establish quality of care in this and other samples.

## Conclusions

This cohort study found evidence that utilization of telehealth services substantially increased following the national public health emergency declaration on March 13, 2020, a time when mental health services were greatly needed. Moreover, service utilization for anxiety, depression, and adjustment disorders gradually increased throughout 2020, although it is likely that the treatment gap for care remained large. It will be important to observe whether and to what extent these trends continue to shift, particularly if and when impermanent legislation to support telemental health services expires or is withdrawn.

## Notes

Supplement.
Data Sharing Statement
