# Supplementary material for: Mental Health Service Utilization Rates Among Commercially Insured Adults in the US During the First Year of the COVID-19 Pandemic
Source: JAMA Health Forum. 2023 Jan 6;4(1):e224936. doi: 10.1001/jamahealthforum.2022.4936 (PMC9857246; doi:10.1001/jamahealthforum.2022.4936)
Supplement: Supplement. — Data Sharing Statement [file jamahealthforum-e224936-s001.pdf]

Data Sharing Statement

McBain. Mental Health Service Utilization Rates Among Commercially Insured Adults in the US During the First Year of the COVID-19 Pandemic. *JAMA Health Forum*. Published January 06, 2023. doi:10.1001/jamahealthforum.2022.4936

Data
Data available: No
